# The biotherapeutic *Clostridium butyricum* MIYAIRI 588 strain potentiates enterotropism of Rorγt^+^Treg and PD-1 blockade efficacy

**DOI:** 10.1080/19490976.2024.2315631

**Published:** 2024-02-22

**Authors:** Thomas Paz Del Socorro, Kentaro Oka, Olivier Boulard, Motomichi Takahashi, Lionel Franz Poulin, Atsushi Hayashi, Mathias Chamaillard

**Affiliations:** a Inserm, University of Lille, U1003, F-59000, Lille, France; b R&D Division, Miyarisan Pharmaceutical Co., Ltd, Saitama, Japan

**Keywords:** Immune checkpoint inhibitors, interferon gamma, interleukin-10, oncomicrobiotic, Rorγt^+^T_reg_

## Abstract

Immune checkpoint inhibitors (ICI) have been positioned as a standard of care for patients with advanced non-small-cell lung carcinomas (NSCLC). A pilot clinical trial has reflected optimistic association between supplementation with *Clostridium butyricum* MIYAIRI 588 (CBM588) and ICI efficacy in NSCLC. However, it remains to be established whether this biotherapeutic strain may be sufficient to heighten the immunogenicity of the tumor draining lymph nodes to overcome resistance to ICI. Herein, we report that supplementation with CBM588 led to an improved responsiveness to antibody targeting programmed cell death protein 1 (aPD-1). This was statistically associated with a significant decrease in α-diversity of gut microbiota from CBM588-treated mice upon PD-1 blockade. At the level of the tumor-draining lymph node, such combination of treatment significantly lowered the frequency of microbiota-modulated subset of regulatory T cells that express Retinoic Orphan Receptor gamma t (Rorγt^+^ Treg). Specifically, this strongly immunosuppressive was negatively correlated with the abundance of bacteria that belong to the family of *Ruminococcaceae*. Accordingly, the colonic expression of both indoleamine 2,3-Dioxygenase 1 (IDO-1) and interleukin-10 (IL-10) were heightened in mice with greater PD-1 blockade efficacy. The CBM588-induced ability to secrete Interleukin-10 of lamina propria mononuclear cells was heightened in tumor bearers when compared with cancer-free mice. Conversely, blockade of interleukin-10 signaling preferentially enhanced the capacity of CD8^+^ T cells to secrete Interferon gamma when being cocultured with CBM588-primed lamina propria mononuclear cells of tumor-bearing mice. Our results demonstrate that CBM588-centered intervention can adequately improve intestinal homeostasis and efficiently overcome resistance to PD-1 blockade in mice.

## GRAPHICAL ABSTRACT

**Figure:**
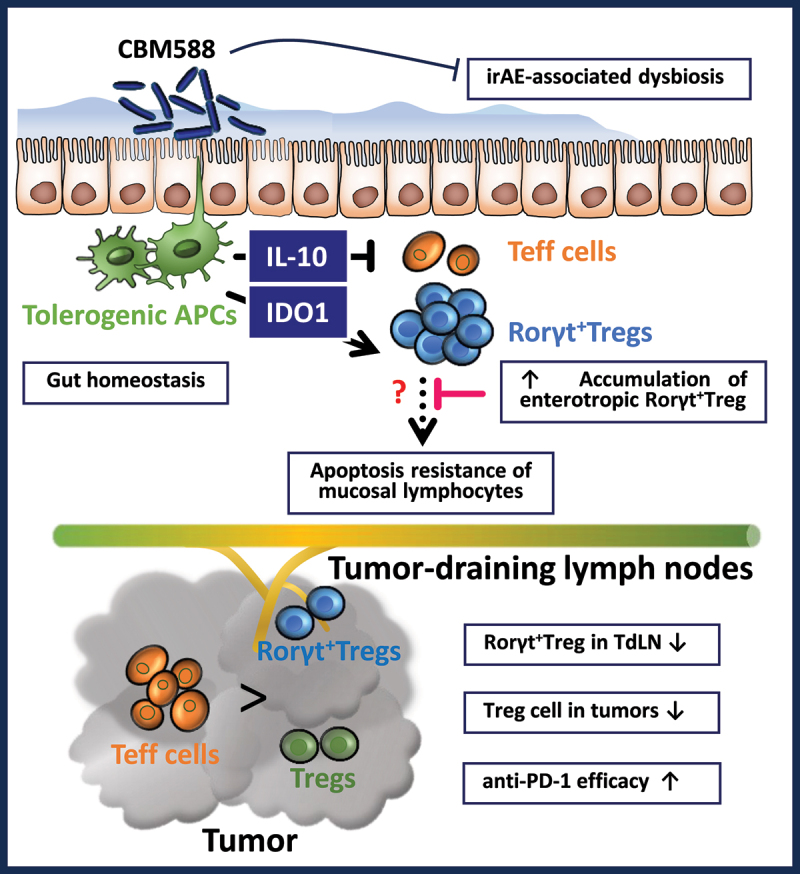

## Introduction

Non-small-cell lung carcinomas (NSCLC) represents about 80% of cases with lung cancer^1,2^ and remains by far the main worldwide cause of cancer-related mortality for either men or women. It is estimated to be responsible for the lowest rate of five-year overall survival with nearly 1.8 million deaths worldwide in 2020. Compared to what is observed with standard chemotherapy, two randomized, controlled trials have provided robust evidence of an impressive improvement of survival rate with Nivolumab in advanced NSCLC.^3,4^ This led to the first regulatory approval for the first-line treatment of metastatic NSCLC by an immune checkpoint inhibitor (ICI) that is targeting the interaction with programmed cell death protein 1 (PD-1). This prescribed medication is a fully human IgG4 monoclonal antibody that targets PD-1. Despite the promising observations with PD-1 blockade in advanced NSCLC, cancer immunotherapy triggers an efficient T-lymphocyte mediated immune surveillance in a minority of patients.^5^ In certain circumstances that still remain poorly understood, a rapid progression is occasionally observed, reinforcing the urgent need to identify ways to efficiently circumvent resistance to ICI. Notably, a primary resistance is observed in about 70 to 80% of cases and a majority of responder patients will ultimately relapse as a consequence of several mechanisms that are either extrinsic or intrinsic to the tumor cell.^6^ Only recently have elegant studiesreported that the ability of anti-PD-1 mAb in lowering lung tumor growth largely depends on specific community structure within gut microbiota at baseline. Accordingly, a worst clinical outcome is observed when administering antibiotics prior to ICI as evidenced by large meta-analyses and prospective trials.^7^

An oncomicrobiotic has been defined as a commensal that may influence cancer immunosurveillance and response to anti-tumor treatment through either direct or indirect suppression the interaction of T lymphocyte inhibitory receptors with their cognate ligands on tumor cells.^8^ In agreement with the possibility that the biotherapeutic *Clostridium butyricum* MIYAIRI 588 (also referred as CBM588) may be considered as a suitable oncomicrobiotic, recent clinical studies have raised the hope that CBM588 may safely improve the response rate to ICI.^9–11^ Indeed, patients with advanced NSCLC who were supplemented daily with CBM588 experienced a longer overall survival rate than those who did not receive CBM588.^9^ Likewise, similar results were observed in patients with NCSLC, even when being treated by proton pump inhibitors.^10^ CBM588 strain is an obligate anaerobic Gram-positive bacterium from cluster I that was isolated in 1960 from soil in Nagano, Japan. Such spore-forming rod-shaped bacteria is prescribed in adequate amounts for the treatment of antibiotic-driven diarrhea in Asia.^12^ In Europe, CBM588 supplementation is authorized as a food additive for improving zootechnical performance in broiler chickens, weaned piglets and turkeys. Devoid of pathogenic markers and clostridial toxin genes, CBM588 supplementation in mice primarily promotes accumulation of induced regulatory T cells (T_regs_) in the colon through induction of Tumor Growth Factor-beta and interleukin-10 (IL-10) by antigen presenting cells.^13,14^ Collectively, this led us to postulate that it may probably conduct a potential causal effect on the conversion of non-responder into responder lung tumor bed. Herein, we provide experimental evidence that live CBM588 could withhold a subset of T_regs_ cells that express the Th17-related transcription factor ROR gamma t (Rorγt^+^ T_reg_) within the colonic mucosa. Consequently, the enhanced response to PD-1 blockade in patients supplemented with a live biotherapeutic CBM588 may be supported by a greater accumulation of immunosuppressive Rorγt^+^ T_reg_ to the large intestine that may contribute to a more immunogenic reprogramming of the tumor-draining lymph node that are strategically positioned for draining the tumor.

## Results

### Oral supplementation of CBM588 improves the efficacy of anti-PD-1 therapy in mice

**Figure 1.**
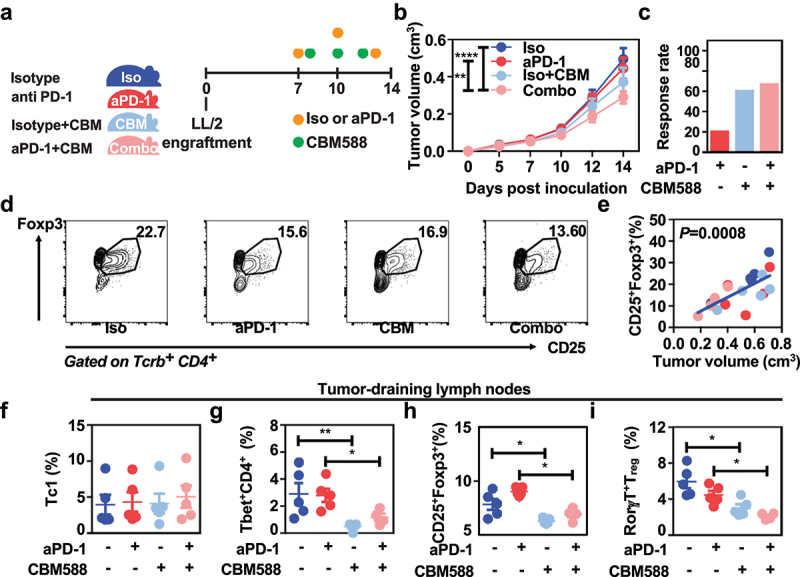
Supplementation with live CBM588 improves the efficacy of PD-1 blockade and lowers the accumulation of RorγT-expressing regulatory T cells at the tumor-draining lymph nodes. (a) Experimental design. Tumor-bearing C57BL/6j mice (n = 10 per condition except for the Combo group in which a mouse with a necrotic tumor was excluded) were treated with either isotype antibody (iso, dark blue line), neutralizing aPD-1 antibody (red line), CBM588 together with isotype antibody (iso + CBM, blue light line) or CBM588 together with neutralizing aPD-1 antibody (Combo, pink line). (b) Tumor growth of two independent experiments. The number of responders per group is indicated between parenthesis and determined as described in material and method section. (c) Response rate to PD-1 blockade. (d) Representative gating strategy in CD25^+^Foxp3^+^ regulatory T cells by flow cytometry analysis. The values correspond to the mean of each experimental condition. (e) Correlation of tumor-infiltrating CD25^+^Foxp3^+^ T_reg_ cells percentage and tumor volume measures in cm^3^ as determined by Caliper. (f) Frequency of interferon gamma-expressing CD8+ T cells (Tc1) in tumor-draining lymph nodes. (g) Frequency of tbet-expressing CD4^+^ cells in tumor-draining lymph nodes, (h) frequency of CD25^+^Foxp3^+^ T_reg_ cells in tumor-draining lymph nodes, (i) frequency of RorγT-expressing T_reg_ cells in tumor-draining lymph nodes. Except for tumor growth, a representative experiment containing 5 mice/group out of two independent experiment is depicted. For panels B, F, G, H, and I, data are plotted as means ± SEM and *p* values were calculated using the Mann-Whitney U test. For (B), a two-way ANOVA is used. p < .05(*), p < .005(**), *p*<,0001 (****).

To assess the impact of live CBM588 on the efficacy of anti-PD-1 therapy in mice, we took advantage of an established syngeneic LL-2 lung adenocarcinoma that leads to the outgrowth of resistant tumor to PD-1 blockade.^15^ The subcutaneous implantation of such poorly immunogenic LL-2 cells give rise to a tumor that reaches a volume of 35 to 50 mm^3^ within seven days. Mice that were treated with either anti-PD-1 mAb or isotype control, were then supplemented or not with live CBM588 every 2–3 days (Figure 1a). In agreement with previous report,^15^ PD-1 specific antibody therapy failed to restrict progression of LL-2 tumor cells when being subcutaneously injected (Figure 1b). The tumor progression was significantly improved in mice that were treated with both PD-1 blockade and CBM588 when compared to what observed in control mice that solely received anti-PD-1 antibodies (Figure 1B and Supplementary Figure S1). At the last recorded measurement of tumor volume, the control of response rate to antibody against PD-1 was enhanced by three-fold in mice that were concomitantly gavaged with CBM588 (Figure 1c). We then tested whether oral administration of CBM588 may locally improve the intestinal toxicity upon PD-1 blockade. To this end, tumor-bearing mice were gavaged with Fluorescein IsoThioCyanate (FITC)-labeled dextran. Fluorescence measurements of serum collected from CBM588-supplemented mice that were concomitantly treated with neutralizing anti-PD-1 antibodies were lowered when compared to control animals even though it failed to reach significance (Supplementary Figure S2A). Accordingly, no changes were detected in intestinal expression of Zonula Occludens 1 that is also known as Tight junction protein-1 (Supplementary Figure S2B). Together, these data demonstrate that oral supplementation with CBM588 is sufficient for improving the efficacy of anti-PD-1 antibodies in mice that are bearing established tumors.

### A lowered intratumoral accumulation of immunosuppressive RORγt^+^ T_reg_ is associated with the enhanced effectiveness upon PD-1 blockade

We next asked whether the heightened efficacy of PD-1 blockade that is induced upon CBM588 supplementation could be attributed to a possible change within the immunosuppressive microenvironment at the tumor bed. At day 14, a cell suspension from each tumor was stained with antibodies that specifically recognize cell surface markers and analyzed with an automated flow cytometer. Interestingly, the tumor growth significantly correlated with the intra-tumoral frequency of the CD25^+^FOXP3^+^ regulatory T cells (T_reg_) (Figure 1d–e). These results suggested us that the decreased intra-tumoral accumulation of T_reg_ in mice that were supplemented with CBM588 may optimally contribute to the lowered tumor growth of mice upon PD-1 blockade. By contrast, no difference in the tumor-draining lymph nodes was observed for the frequency of CD8^+^ T cells that are known for their capacity to secrete IFN-γ, granzyme and perforine (Figure 1f). Furthermore, we found that the proportion within the tumor-draining lymph nodes of both Tbet-expressing CD4^+^ T cells and T_reg_ was significantly lowered in response to the combination of treatment when compared to controls or PD-1 blockade alone (Figure 1g–h). Among the heterogeneous population of naturally occurring T_reg_, we noticed a lowered frequency of a small subset of Rorγt-expressing T_reg_^16^ within the tumor-draining lymph node in response to the combination of treatment as depicted in Figure 1i. Unlike T_reg_ cells that are thymically derived, this unusual subset of T_reg_ cells (that do not express Helios or Neuropilin-1) has a tropism to the colon is induced in the colon by antigen presenting cell in a CCR7-dependent manner.^17^ By contrast, none of the treatments were found to induce some significant differences on the splenic frequency of either effector or regulatory T cells (Supplementary Figure S3A-D). Given that the majority of intra-tumoral T_reg_ cells failed to express Rorγt, it is unlikely a CBM588-modulated signals may cause Rorγt-expressing T cells to home to tumor. Instead, these data support the possibility that sensing of CBM588 may actively instructs this enterotropic subset of T_reg_ within the colonic mucosa for promoting the establishment of a tumor-draining lymph node microenvironment that is sensitive to PD-1 blockade.^18,19^

### The beneficial impact of CBM588 on the effectiveness of PD-1 blockade is linked to a lowered richness of the gut microbiota

**Figure 2.**
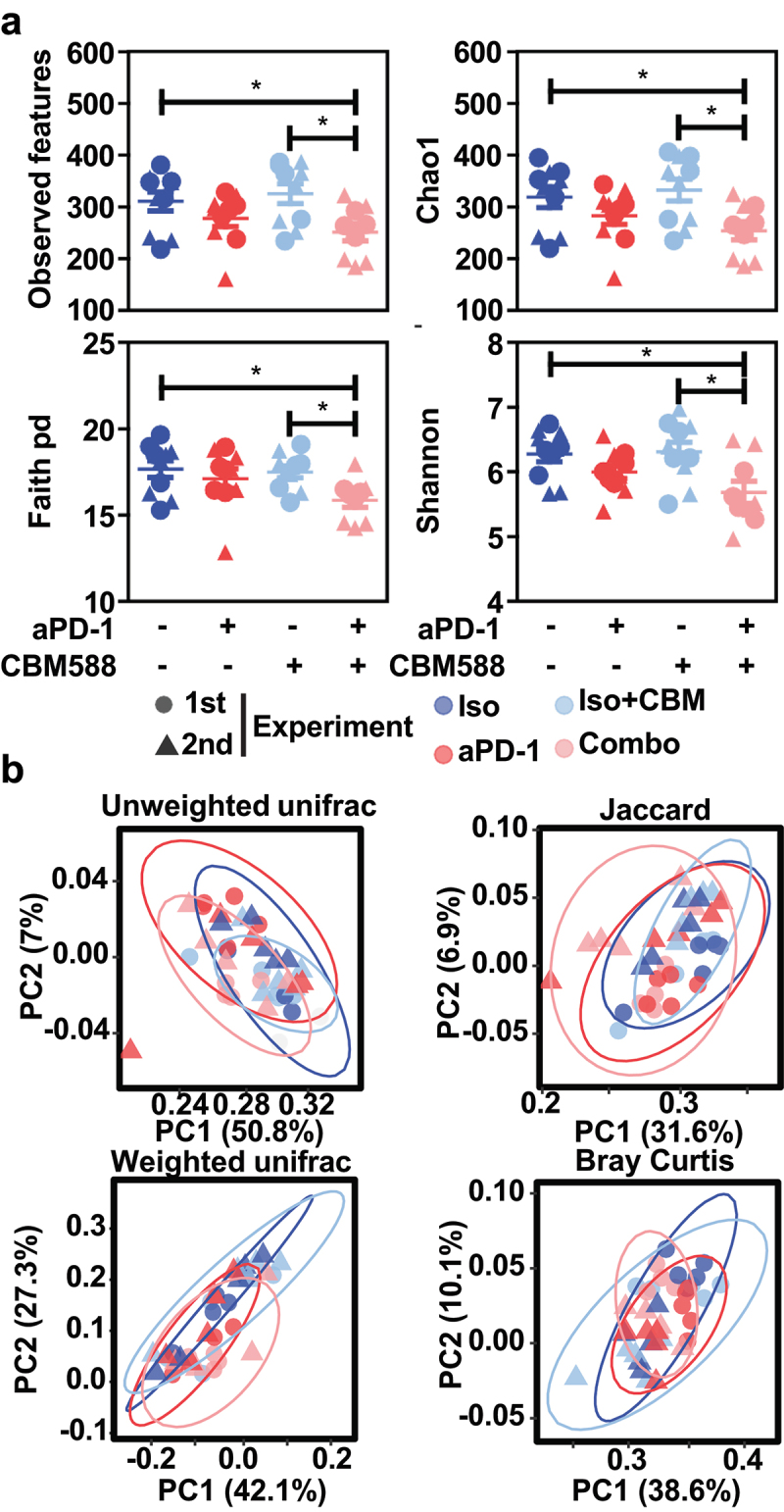
The CBM588-induced response to PD-1 blockade is linked to a reduced alpha diversity of the gut microbiota. (a) boxplots representing multiple metrics of alpha diversity at day 14 from mice that did not receive antibiotics. (b) PCoA plots of several metrics of beta diversity at day 14 from mice that did not receive antibiotics treated mice. *p* values from the Mann-Whitney U test with Benjamini-Hochberg adjustment are indicated in panel A.

Several recent clinical studies revealed a link between the reduced alpha diversity and the anti-PD1 response rate in patients with lung cancer.^20^ We next asked whether the improved anti-tumor immune response in mice treated with CBM588 is associated with specific changes in the fecal composition of bacteria. To this end, the structure and composition of bacterial communities was surveyed by 16S rRNA gene sequencing of fecal DNA. A range of 36,062–90,091 quality filtered reads per sample were generated for subsequent Qiime 2 analysis. Commonly used measures of alpha diversity were next inspected for qualitatively determining whether some treatment may be responsible for differences in individual composition of the fecal microbiota. Non-parametric Kruskal–Wallis tests was then used to reveal significant differences in community heterogeneity and Mann–Whiteny U test with Benjamini–Hochberg false discovery rate (FDR) correction was used to account for multiple testing, if any. Even though CBM588 does not persist within the intestine,^21^ several metrics of alpha diversity were significantly lowered within each sample that were collected upon PD-1 blockade in combination with CBM588 when compared to those taken from isotype-treated animals (Figure 2a). Even though the evenness is similar between each experimental condition (Figure 2a), this reduced richness led us to evaluate whether the partitioning of bacterial diversity may somehow vary between our experimental conditions. The aforementioned results led then us to postulate that modulation of bacterial community structure by CBM588 may probably remodel the microenvironment of the tumor-draining lymph nodes to overcome resistance to PD-1-based immunotherapy. This said, the modulation of the richness and/or the evenness of bacterial community structure by CBM588 might turn out to be a way for presumably reshaping the microenvironment of the tumor-draining lymph nodes. We next investigated to what extent the compositional structure may vary upon PD-1 blockade between mice that were supplemented or not with CBM588. To this end, Unweighted and Weighted UniFrac measures were then applied to the same data set as a qualitative and quantitative phylogenetic measure of community beta diversity, respectively. Despite a reduced richness, the visualization by principal coordinate analysis (PCoA) of the UniFrac distance metrics failed to identify significant treatment-induced shift in the microbiota composition (Figure 2b). Similar results were obtained when calculating other quantitative and qualitative measures of beta diversity, which are the Bray Curtis and Jaccard indices, respectively (Figure 2b). These results led us to suggest that the resistance to PD-1 blockade may be linked to the presence or absence of some tolerogenic or immunogenic bacteria in the gut microbiota, respectively.

### Dysbiosis induced by PD-1 blockade is improved upon CBM588 supplementation

**Figure 3.**
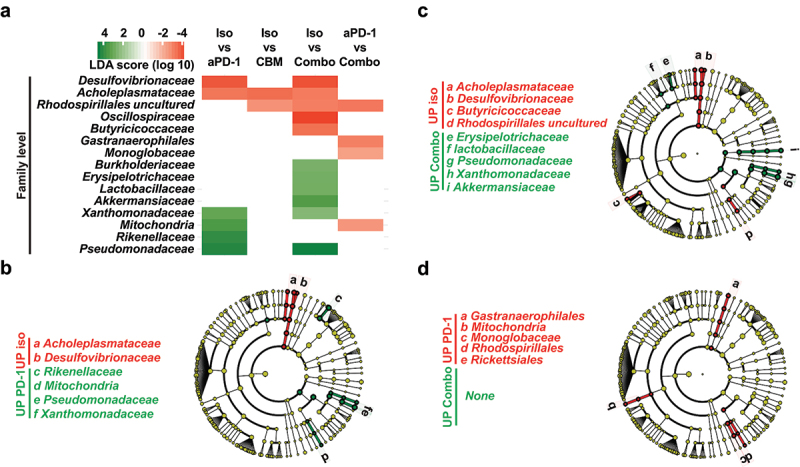
The dysbiosis upon PD-1 blockade is improved upon supplementation with CBM588. LEfSe analysis at several taxonomic level revealed enrichment of multiple bacterial taxa in isotype control or anti-PD-1-treated mice when compared to those who received the combination of treatment. (a) Data are shown as log10 LDA score on each bacterial taxa of which relative abundance is enriched in group a (blue) vs group B (red). (B-D) LEfSe cladograms of differentially abundant bacterial taxa based on pairwise analysis. (b) isotype control vs anti-PD-1. (c) isotype control vs combo. (d) anti-PD-1 vs combo.

Even though a similar alpha and beta diversity was observed between responder and non-responder mice (Supplementary Figure S4), high-dimensional class comparisons between specific taxa were performed by applying Linear discriminant analysis (LDA) Effect Size (LEfSe). LEfSe analysis with a threshold score of 2 revealed that the abundance of eleven genera was changed upon PD-1 blockade (Figure 3a). Notably, a greater predominance of Gram-negative *Pseudomonas* belonging to the family *Pseudomonadaceae* in the class *Gammaproteobacteria* coincided with an increased abundance of genus *Stenotrophomonas* [family *Xanthomonadaceae*, order *Xanthomonadales*, class *Gammmaproteobacteria*)] and genus *Allistipes* [family *Rikenellaceae*, order *Bacteroidales*, class *Bacteroidia*] (Figure 3a). This might be caused by the toxicity of the anti-PD-1 antibodies as what observed in cancer patients with immune-related adverse events (irAE).^22^ Previous work reported that pro-inflammatory bacteria belonging to the family *Desulfovibrionaceae* were more prevalent in feces from patients with renal cancer.^23^ Concomitantly, the frequency of uncultured bacteria from the genus of *Desulfovibrionaceae* was significantly reduced upon PD-1 blockade alone or in combination with CBM588 when compared to what observed in control mice (Figure 3a, b). This is in agreement with the exploratory subgroup analysis that showed a statistically significant decrease of genus *Desulfovibrio* spp. in CBM588 responder group.^11^ In addition to this, the abundance of uncultured bacteria belonging to the family of both order *Rhodospirillales* and order *Gastranaerophilales* that are among the most enriched genera of bacteria upon acute intestinal inflammation was significantly lowered with the combination of treatment when compared with anti-PD-1 alone (Figure 3a, d). Of note, the abundance of *Rikenellaceae* that belong to the same order was enhanced upon PD-1 blockade but not in response to the combination of treatment (Figure 3a–c). Consistent with a previous report showing that *Akkermansia* was linked to a clinical benefit of ICI in patients with NSCLC,^20^ the combination of treatment significantly enhanced the abundance of family *Akkermansiaceae* when compared with isotype mice (Figures 3a & 3c). We then hypothesized that the shift in the gut microbiota composition from mice that were supplemented by CBM588 may be linked to the paucity of enterotropic Rorγt-expressing T_reg_ at the tumor-draining lymph nodes.

### The lowered frequency of Rorγt-expressing T_reg_ cells at the tumor-draining lymph nodes is linked to microbiota-modulated tryptophan catabolism upon supplementation with CBM588

**Figure 4.**
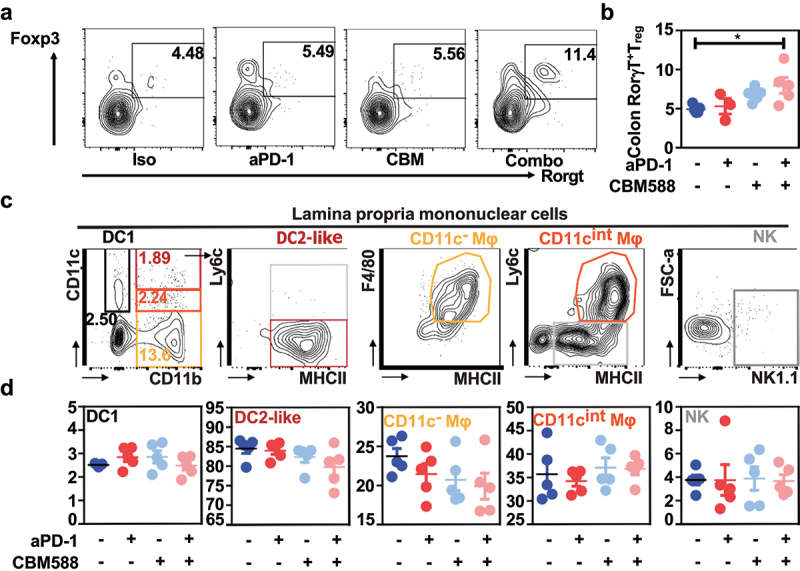
Supplementation with live CBM588 markedly promotes accumulation of RorγT-expressing treg cells in the colon while having no impact on the myeloid compartment.IS9855 the intestinal response to CBM588 was evaluated in the intestine of mice that were treated with either isotype antibody (iso, dark blue dot), neutralizing anti-PD-1 antibody (red dot), CBM588 together with isotype antibody (Iso+cbm, blue light dot) or CBM588 together with neutralizing anti-PD-1 antibody (aPD-1+CBM, pink dot). (a) Representative contour plots representing RorγT-expressing T_reg_ cells in the colon. (b) Abundance of RorγT-expressing T_reg_ cells in the colon. (c) Gating strategy for myeloid cells subset in LPMC. (d) Enumeration of myeloid cells subsets’ frequencies within LPMC. For panels B and D, data are plotted as means ± SEM and *p* values were calculated using the Mann-Whitney U test. For B and D, a two-way ANOVA is used. p < .05(*).

**Figure 5.**
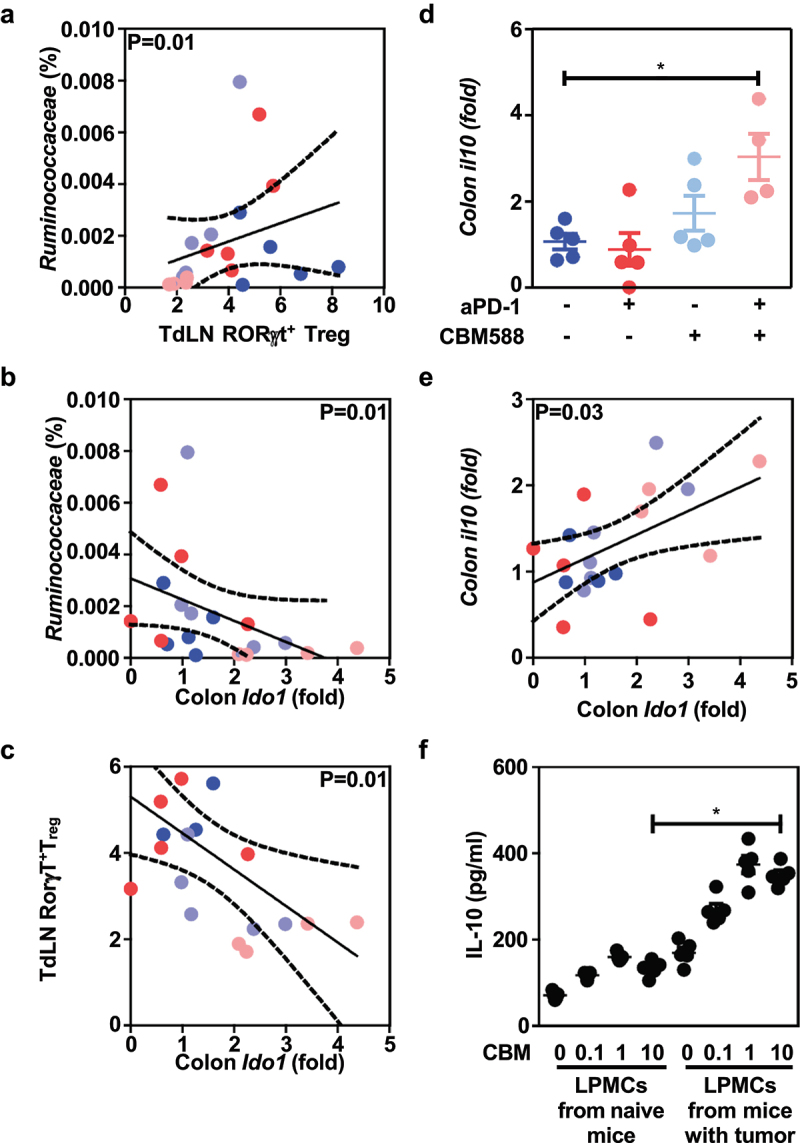
The CBM588-induced immunogenic conversion of the tumor draining-lymph nodes is linked to the IDO1/IL-10 axis upon PD-1 blockade. (a) Spearman correlations between the frequency of RorγT-expressing T_reg_ cells at the tumor bed and the relative abundance of uncultured bacteria that belong to the family of *Rumicoccacea*. (b) Spearman correlations between the transcript level of Ido1 at the colon and the relative abundance of uncultured bacteria that belong to the family of *Rumicoccacea*. A representative experiment with 5 mice per group out of two yielding similar results of RT-qPCR analysis is depicted. (c) Spearman correlations between the transcript level of Ido1 at the colon and the frequency of RorγT-expressing T_reg_ cells at the tumor draining lymph nodes. (d) Normalized transcript level of Ido1 from distal colon tissue by qRT-PCR analysis. Each dot represents a biological replicate (*n* = 5/group). (e) Spearman correlations between the transcript level of Ido1 and Il10 at the colonic mucosa accordingly to legend colors. (f) Quantitative measurements of IL-10 in the supernatant of heat-killed CBM588 at different doses were added to LPMC that were isolated from the colon of mice that beard or not tumor P values were calculated using the Mann-Whitney U test and the Spearman correlation test.

Given that Rorγ^+^T_regs_ reside in greater proportion within the colon when compared to the ileum^18^ (Supplementary Figure S5), we next asked whether the heightened efficacy of PD-1 blockade that is induced upon CBM588 supplementation could be attributed to a possible change within the immunosuppressive microenvironment at the tumor bed. Accordingly, the improved efficacy of PD-1 blockade that is induced by CBM588 was accompanied with a greater frequency of Rorγ^+^T_regs_ within the colonic mucosa (Figure 4a–b). By contrast, no apparent effect was noticed on the frequencies of each main subsets of intestinal mononuclear phagocytes that are one of the most abundant immune cell types in the gut (Figure 4c–d). While this dominant colonic subset of Rorγ^+^T_regs_ are absent within the lamina propria of germ-free animals^18^ or in response to antibiotics,^24^ its frequency in the tumor-draining lymph nodes was found to be only correlated with the relative abundance of uncultured bacteria that belong to the family of *Ruminococcaceae* (Figure 5a). Given that the aforementioned bacteria have the capacity to convert tryptophan, we reasoned that supplementation of CBM588 may subsequently modulate the impact of tryptophan metabolism on the conversion of T_regs_ into T_H_17-like T cells.^25^ Accordingly, the frequency of *Ruminococcaceae* and the paucity of Rorγ^+^T_regs_ within the tumor-draining lymph nodes were both inversely correlated to the colonic transcript level of indoleamine 2,3-Dioxygenase 1 [IDO-1] (Figure 5b–c). This is of particular importance as IDO-1 is the rate-limiting enzyme in the degradation of dietary tryptophan that directly activate mature Tregs.^26^ By contrast, we failed to observe any significant differences in either gene encoding for Mucosal vascular addressin cell adhesion molecule 1 (referred as *Madcam-1*) or other genes that are involved in intestinal induction of Rorγ^+^T_regs_ (Supplementary Figure S6). This is in agreement with a previous report showing that colonic Rorγ^+^T_regs_ did not secrete detectable IL-17a or f.^18^ Together, we reasoned that the lowered accumulation at the tumor bed of Rorγ^+^T_regs_ could result from a decreased catalytic conversion of tryptophan to the immunosuppressive metabolite kynurenines, which engage Aryl Hydrocarbon Receptor signaling for balancing mucosal reactivity and promoting expansion of enterotropic T_regs_ and their transcriptional regulation.^27^

### The CBM588-induced immunogenic conversion of the tumor draining-lymph nodes is linked to the IDO1/IL-10 axis upon PD-1 blockade

**Figure 6.**
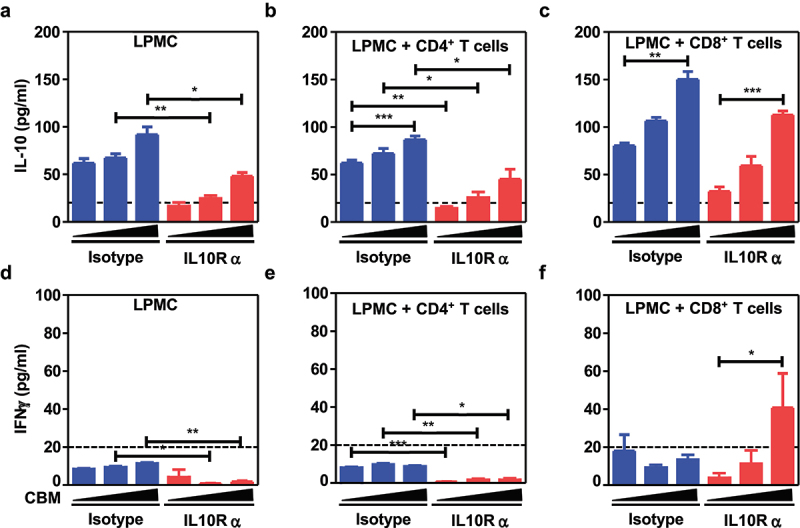
CBM588 heightened IL-10-mediated prevention of overproduction of interferon gamma by CD8+ T cells. Quantitative measurements of interleukin-10 in the supernatant from LPMCs from tumor bearers (a) and cocultures with either CD8^+^ T cells (b) or CD8^+^ T cells (c) upon or not blockade of IL-10 signaling. Quantitative measurements of interferon-gamma in the supernatant from LPMCs from tumor bearers (A) and cocultures between LPMCs (d) and CD4^+^ T cells (e) and CD8^+^ T cells (f) upon or not blockade of IL-10 signaling. As indicated in the figure 6, cocultures between LPMCs and CD4^+^ T cells or CD8^+^ T cells were treated with either heat-killed CBM588 (at multiplicity of infection of 0.1 or 1). Data are plotted as means ± SEM and *p* values were calculated using the Mann–Whitney U test. For (C) a two-way ANOVA is used. p < .05(*), p < .005(**), p < .0005(***).

This led us to further explore how supplementation with CBM588 may significantly enhance colonic expression of IDO-1 when combined with anti-PD1 antibodies (Figure 5d), which is closely linked with several pathways that modulate T cell-mediated anti-tumoral immunity. Among those, there was a strong correlation between the transcript level of IDO-1 and Interleukin-10 (Figure 5e). This is in agreement with the observation that heat-killed CBM588 is able to induce IDO-1 expression (Supplementary Figure S7). Given that it has been established that CBM588 can cause the production of biologically active Interleukin-10 by intestinal mononuclear phagocytes,^13^ we then examined whether lamina propria mononuclear cells (LPMCs) may secrete such immunosuppressive factor when being stimulated with heat-killed CBM588 in a dose-dependent manner. Given the ability of IL-10 to potentiate the efficacy of PD-1 blockade in NSCLC,^28^ we then reasoned that the greater capacity of intestinal mononuclear phagocytes to secrete the immunosuppressive IL-10 cytokine in response to CBM588 may probably contribute to the greater responsiveness to anti-PD1 antibodies. Accordingly, the secretion of IL-10 by LPMCs was significantly heightened by heat-killed CBM588 in a dose-dependent manner (Figure 5f). Similar results were expectedly obtained with either bone-marrow derived macrophages or bone-marrow derived dendritic cells (Supplementary Figure S8). As depicted on panel F of Figure 5, such property of heat-killed CBM588 was further enhanced when LPMCs were isolated from tumor-bearing mice. Similar findings were observed when LPMC were isolated from the colon of tumor bearers (Figure 6a). By contrast, the ability of LPMC to secrete IL-10 was not modulated in response to the supernatant of CBM588 (Supplementary Figure S10), suggesting that sensing of some cell wall components from CBM588 by intestinal phagocytes may promote the secretion of interleukin-10 for dampening the production of interferon-gamma by CD8^+^ T cells. This is in agreement with the capacity of CBM588 to directly induce IL-10 production by macrophages via the TLR2/MyD88 pathway.^13^

### The inhibitory effect of CBM588 on CD8 cytotoxic T cells is IL-10 dependent

One hypothesis is that sensing of CBM588 may promote resistance to apoptosis of tumor-specific CD8^+^ T cells to a similar extent as what observed when mice with advanced tumors receive Cetuximab-based IL-10 fusion protein.^29^ In agreement with this possibility, qRT-PCR analysis revealed a similar gene expression profile with the tumor of mice that were treated or not with CBM588 upon PD-1 blockade (Supplementary Figure S9). This led us to further investigate whether CBM588-induced IL-10 may locally inhibit interferon gamma (IFNγ) secretion by either CD8^+^ or CD4^+^ T cell. To this end, LPMC from tumor bearers were cocultured with CD8^+^ or CD4^+^ splenocytes in the presence of blocking IL-10 R Ab 1B1.3a or its isotype. Treatment with CBM588 of LPMC that were cocultured with CD8^+^ splenocytes significantly heightened the secretion of IFNγ upon IL-10 signaling blockade (Figure 6f). By contrast, the level of IFNγ was barely detectable in the supernatant of either LPMC alone or in coculture with CD4^+^ T cells despite similar amounts of IL-10 (Figure 6d–e). Concomitantly, the level of IL-10 upon blockade of IL-10 signaling was markedly lowered when compared to isotype (Figure 6a–c). This accords with the previously reported autocrine regulatory loop that modulates the immunomodulatory activity of macrophages. Future experimentation should address whether CBM588 may modulate directly or indirectly the establishment and maintenance of the macrophage niche within the intestinal mucosa.

## Discussion

It is now established that the response rate to ICI is often impaired in patients who received antibiotic therapy.^7^ Herein, we provide the proof-of-concept that CBM588 might be an effective and safe live biotherapeutic bacterial strain for immunogenic conversion of the tumor-draining lymph nodes and subsequent improvement of its responsiveness to ICI. Unlike what is observed in the small intestine, the majority of this immunosuppressive subset of T_reg_ express the transcription factor FoxP3 in the colon.^24^ Within the highly heterogeneous population of T_reg_, it has been established that a discrete subset that express the Th17-related transcription factor Rorγt acquired attributes for promoting cancer.^16^ In the herein particular setting of ICI resistance that is characterized by an unrepressed increase in production of IFN-gamma (Supplementary Figure S9), the supplementation with CBM588 significantly decreased the frequency of Rorγ^+^T_regs_ within the tumor draining lymph nodes at day 14 (but not in the tumor). The possibility that the CBM588-derived metabolites may alter the maturation and/or the exodus of Rorγ^+^T_regs_ within the colonic lamina propria warrants further investigation. Specifically, fate mapping studies using reporter mice could offer insights into when and where antigen presenting cells may be locally primed by CBM588 for expanding Rorγ^+^T_regs_ and preventing their migration from the large intestine to the tumor (Graphical abstract). While being found more abundant within the colonic mucosa of mice treated with Combo, this immunosuppressive subset is naturally retained within the colonic mucosa for homeostasis through a variety of mechanisms such as Tryptophan metabolites.^27^ Accordingly, it was accompanied by a lowered abundance of bacteria that have the capacity to convert tryptophan. This led us to support a model in which the CBM588-induced secretion of interleukin-10 by antigen-presenting cells may be responsible for the increased expression of IDO-1 in the colon that locally facilitate the expansion of RORγt^+^ Treg cells^27^ (Graphical abstract). Given that we observed a lowered frequency of Rorγ^+^T_regs_ at the tumor bed (Figure 1i), we then reasoned that CBM588 supplementation could facilitate tryptophan catabolism for limiting the exodus of activated RORγt^+^ Treg cells at the tumor-draining lymph nodes. Accordingly, dietary tryptophan deficiency reshapes the microbiota and alters intestinal gene expression profiles, resulting in a microbiota-dependent expansion of gut RORγt^+^ Treg.^27^ Even though it remain to be evaluated, one may then anticipate a decreased frequency of Gata3^+^ Treg cells that are gerenated at the expense of Rorγ^+^T_regs_ as what observed upon dietary tryptophan deficiency.^27^ Given that activated T cells are sensitized to apoptosis upon tryptophan deprivation,^30^ this event may allow immunogenic conversion of the microenvironment of the tumor-draining lymph nodes for preventing interferon gamma-mediated antigen-specific CD8^+^ T cell apoptosis. It is then conceivable that the accumulation of Rorγ^+^T_regs_ within the intestine of mice that were supplemented with CBM588 may at least partly contribute to their greater responsiveness to anti-PD-1 antibodies and to the improvement of intestinal toxicity. It follows previous demonstration on the significance of migratory and T_regs_ expanding molecules as selective targets for preferential retention of Rorγ^+^T_regs_ within the colonic mucosa.^31^ This said, it is worth noting that CBM588 supplementation failed to modulate the transcript level of Madcam-1 in the colon even in response to PD-1 blockade (Supplementary Figure S6). By contrast to what observed in response to *Akkermansia*^*19*^, this suggests that CBM588 may unlikely promote the colonic expansion of Rorγ^+^Treg cells through a mechanism that relies on MAdCAM-1. Interestingly, it has been established that IL-10 prevented the trafficking of intestinally derived CD4^+^ T cells to the liver.^32^ Given that CD25^+^ Foxp3^+^ T_reg_ cells can be reprogrammed into a pathogenic subset within an inflammatory settings,^33^ these data raise the possibility that the improvement of intestinal toxicity upon supplementation with CBM588 may prevent such pathogenic conversion through IL-10 for improving effector CD4^+^ T cell-mediated response that is required for optimal response to anti-PD-1 antibodies. This accords with previous report describing a capacity of CBM588 to alleviate the severity of intestinal damage by promoting IL-10 by intestinal phagocytes in different preclinical models.^13,34,35^ Under this specific circumstance, the greater ability of LPMCs to secrete IL-10 in response to CBM588 support the idea that such tolerogenic effect was associated with a lowered expansion of Rorγ^+^T_regs_ at the tumor-draining lymph nodes. This led us to further investigate whether CBM588-modulated interleukin-10 signaling in T cells may enhance their capacity to secrete interferon gamma for promoting the efficacy of PD-1 blockade. In agreement with this hypothesis, a lowered secretion of interferon gamma by CD8^+^ T cells was measured when being cocultured with CBM588-primed LPMCs (Figure 6). It coincided with a greater frequency of *Ruminococcaceae* that may presumably modulate the impact of tryptophan metabolism on the conversion of T_regs_ into T_H_17-like T cells.^25^ This accords with the improvement of colitis severity upon CBM588 supplementation that is maintained in T_reg_-depleted animals and mice that lack functional T and B cells.^36^ Concurrent with the acquisition of a regulatory phenotype of intestinal phagocytes, the anti-inflammatory property of CBM588 on DSS-induced colitis is lost in macrophage-specific IL-10-deficient mice.^13^ This said, there are limitations in study design that may have influenced our understanding of the mode of action of CBM588. Notably, our study cannot ruled out the possibility that supplementation CBM588 may modulate resilience of the gut microbiota upon PD-1 blockade. Furthermore, one may reason that patients’ diet may have influenced the composition of their gut microbiota in the setting of treatment with ICI. Indeed, 16S rRNA sequencing failed to reveal differences across multiple diversity metrics in NSCLC patients that were supplemented with CBM588.^10^ Given that supplementation with CBM588 improved antibiotic-induced dysbiosis,^37^ it remains to be determined whether IL-10 signaling is responsible for the lowered abundance of uncultured bacteria that belong to the family of *Ruminococcaceae* and subsequently to the CBM588-induced improvement of intestinal barrier function.^37^ Collectively, the potential ability of CBM588 in heightening the efficacy of ICI may depend on the acquisition of a regulatory phenotype of intestinal phagocytes that limits intestinal damage and accumulation of Ror^+^T_regs_ at the tumor-draining lymph nodes upon PD-1 blockade (Graphical Abstract).

## Materials and methods

### Mice

Wild-type C57BL/6J female mice at 4 weeks of age were obtained from the specific opportunist pathogen-free (SOPF) breeding facility of Janvier (France). Animals were housed up to five per cages and had free access to a standard laboratory chow in SOPF conditions at the *Institut Pasteur de Lille*.

### Culture of LL/2 cells and generation of bone-marrow-derived dendritic macrophages

The Lewis lung carcinoma cells (LL/2) LL/2 cell line that is syngeneic for C57BL/6 mice (ATCC CRL-1642™) was purchased from ATCC as a model to study human lung cancer that is resistant to PD-1 blockade. The aforementioned tumor cell line was cultured at 37°C under 5% CO_2_ complete medium Dulbecco’s Modified Eagle Medium (DMEM), High Glucose and Glutamax, which is supplemented with 10% heat-inactivated fetal bovine serum (FBS), 100 units/ml penicillin G sodium, 100 μg/ml streptomycin sulfate, 2 mM L-glutamine, 1 mM sodium pyruvate and non-essential amino acids (all reagents from Gibco). Bone marrow-derived macrophages (BMDMs) were isolated from femurs of wild-type mice of C57BL/6J background. Using a 26 G ½” needle, bone marrow cells were flushed out of the bones with Iscove’s modified Dulbecco’s medium (IMDM), supplemented with 10% fetal bovine serum, 1% penicillin/streptomycin, 1% non-essential amino acid, 1% sodium pyruvate, 1% glutamine, and 20% (v/v) conditioned-media from L929 cells. Red blood cells were lysed using a 160 mM NH4CL and 170 mM Tris solution for 5 min at RT. Three to six × 10^6^ viable cells were plated in non-cell-culture-treated petri dishes and grown for 5–7 d in the above mentioned fully supplemented IMDM medium.

### Supplementation with CBM588 spores

The biotherapeutic *Clostridium butyricum* MIYAIRI 588 (CBM588) was obtained from Miyarisan Pharmaceutical. Age-matched tumor-bearing mice were inoculated three times per week by oral gavage with 100 μl of suspension containing 1 × 10^8^ CFUs of CBM588 spores on the day following the first anti-PD-1 injection.

### Tumor challenge and treatment with neutralizing anti-PD-1 antibody

Mice were subcutaneously injected into the right flank with 5 × 10^5^ tumor cell line (*n* = 5 per group). When tumors reached a size of 20 to 40 mm^3^, mice were then injected intraperitoneally (i.p) at 3-day intervals with 250 μg of anti-PD-1 mAb (clone RMP1–14, BioXcell, NH, USA) or isotype control (clone MPC11). The experimental group were the following: 1) Isotype, 2) anti-PD-1 treatment, 3) Isotype and CBM588, 4) anti-PD-1 treatment and CBM588. Tumor growth over two-weeks were routinely monitored by means of a caliper. Briefly, measurements in mm of two perpendicular diameters using a Caliper were used every 2–3 days as a metric of subcutaneous tumor volume for monitoring tumor growth calculated according to the formula largest diameter multiplied with smallest diameter^2^/2. Mice were classified as responder when the specific growth rate (% increased volume at day 14 when compared at day 7 before initiation of PD-1 blockade) of their tumor was decreased by more than 40% when compared to the median of specific growth rate of tumors from the group of mice that received the isotype. Experiments were performed at least twice.

### FITC-dextran assay to assess intestinal permeability

Two days following their last i.p. mAb administration, mice were water-starved overnight. The following day, mice were orally administered with 0.44 mg/g body weight of a 100 mg/ml solution of FITC-dextran (FD4, Sigma) in PBS (pH 7.4). Four hours later, blood was collected from each mouse by retro-orbital sampling. Blood was allowed to clot overnight at 4°C, then subsequently centrifuged at 3,000 rpm for 20 min to collect the serum. Dilutions of FITC-dextran in pooled mouse serum from naïve mice were used as a standard curve together with serum from mice that did not receive FITC-dextran to remove the background. The absorbance of 100 μL serum (diluted in PBS) was measured by an automated microplate reader with excitation and emission filters set at 485 nm (20 nm band width) and 528 nm (20 nm band width), respectively.

### Feces collection and DNA extraction

Approximately 30 mg of feces were collected at the end of tumor growth for subsequent 16S rRNA analysis and kept frozen until analysis. The collected samples of feces were suspended in 250 μL of a solution that contains 4 M guanidine thiocyanate, 100 mM Tris-HCl [pH 9.0], and 40 μL of N-Laurosyl Sarcosine 10%. When homogenized, a volume of 500 μL of Phosphate 0.1 M was added to the suspension. The resulting suspension was then incubated at 70°C for 1 h After one hour at 70°C, about 500 mg of glass beads with a diameter of 0.15–0.21 mm were added and followed by beating with a MagNA Lyser (Roche) operating at 25 Hz for 10 s. After bead fractionation, 15 mg of polyvinylpolypyrrolidone was added before being centrifugation of the suspension for 5 min at 20,000 × g. The pellet was then resuspended in 500 μl of TENP (50 mM Tris-HCl pH 8.0, 20 mM EDTA, 100 mM NaCl and 0.01 g/ml polyvinylpyrrolidone). After the addition of a volume of 1 mL of ice-cold isopropanol, the tubes were inverted several times to mix the contents before being left 10 min at room temperature. The tubes were then centrifuged at 20,000×g for 10 min, and the resulting supernatants were discarded by decanting. The remaining DNA pellets were dissolved in 450 μL of a solution based on phosphate buffer pH8 at 0.1 M with potassium acetate at 5 M. After overnight incubation at 4°C, the tubes were then centrifuged at 20,000×g for 30 min at 4°C. A volume of 2 μL of RNase at 10 mg/mL was then added to the supernatant before being incubated for 30 min at 37°C. A volume of 1 mL of absolute ethanol to which was then added with 50 μL of sodium acetate at 3 M before a 20,000×g centrifugation for 10 min at 4°C. This washing step was then repeated two times before proceeding to a resuspension with 50 μL of TE 1X.

### Gene expression analysis

The RNA was extracted from colon that were sampled at day 14. Colonic RNA was then reverse-transcribed with the AffinityScript QPCR cDNA Synthesis Kit according to the manufacturer’s instructions (Agilent Technologies). The resulting cDNA (equivalent to 5 ng of total RNA) was amplified using the SYBR Green real-time PCR kit and detected on either MxPro or AriaMx (Agilent Technologies). RT-qPCR analysis was performed with the forward and reverse primers that were designed using Primer 3 software (sequences available upon request). On completion of the qPCR amplification, a DNA melting curve analysis was carried out in order to confirm the presence of a single and specific amplicon. *Actb* were used as an internal reference gene in order to normalize the transcript levels of each genes of interest. Relative mRNA levels (2-2−ΔΔCt) were determined by comparing (a) the PCR cycle thresholds (Ct) for *Actb* and the genes of interest (ΔCt) (b) ΔCt values for treated and control groups (ΔΔCt).

### Tissue dissociation, enzymatic digestion for preparing cell suspension and isolation of lamina propria mononuclear cells and splenic CD4^+^ T cells and CD8^+^ T cells

Tumor-draining lymph nodes and spleens were harvested 2 days after the third injection of anti-PD-1. Excised lymph nodes and spleens were teased apart into a single cell suspension by pressing with the plunger of a 3 ml syringe in RPMI medium. Tumor were dissociated into small pieces and digested in RPMI medium containing Collagenase IV (0.625 μg/mL, Sigma-Aldrich) and DNase1 at 150UI/ml (Roche Diagnostics) for 30 min at 37°C. The mixture was subsequently passage through a 70 μm cell strainer. After isolation, cells were passed through a 40 μm cell strainer before use (BD biosciences). CD4^+^ T cells were isolated from the spleen of tumor-bearing C57BL6/J mice by negative depletion of non CD4^+^ T cells with anti-biotin MicroBeads according to manufacturers’ protocol (Miltenyi Biotec). The negative fraction was used to isolate CD8^+^ T cell with a negative selection kit (Miltenyi Biotec). The purity of over 95% CD4^+^ T cells and CD8^+^ T cells was confirmed by flow cytometry characterization of magnetic-sorted cells. Enzymatic digestion of the intestine was performed for isolating lamina propria mononuclear cells (LPMCs) from tumor-bearing mice that were supplemented or not with CBM588 upon PD-1 blockade or not as previously described.^38^ Briefly, cells were isolated from colons, after removal of epithelial cells, by enzymatic digestion with 1.25 mg/ml collagenase D (Roche Diagnostics), 0.85 mg/ml collagenase V (Sigma-Aldrich), 1 mg/ml Dispase (Life Technologies), and 30 UI/mL DNaseI (Roche Diagnostics) in complete RPMI 1640 for 30–40 min in a shaking incubator until complete digestion of the tissue. The procedure of digestion has been optimized for the ileum by replacing the collagenase D with the collagenase type VIII (Sigma) at a concentration of 1.25 mg/mL.

### Flow cytometry analysis

Tumor cells were pre-incubated with purified anti-mouse CD16/CD32 (clone 93; eBioscience) and normal mouse serum (Interchim, UP379121) for 15 min at 4°C, before membrane staining. Dead cells were excluded from the FACS analysis using the Live/Dead Fixable Violet dead cell stain kit (L34955 Thermo Fischer). The FACS analysis of effector and regulatory T cells was performed by making use of the following antibodies CD45-BV605 (30-F11, Biolegend 1/200), CD4-Viogreen (REA1211, Miltenyi 1/200), CD8a-PEVio770 (REA601, 1/200, Miltenyi), TCR-beta-FITC (H57–597, Biolegend, 1/200), CD25-PEVio770 (REA568, Miltenyi, 1/200), t-bet-PE (4B10, Biolegend, 1/200), Foxp3-PE (REA788, Miltenyi, 1/200), RorγT-PECF594 (Biosciences, Q31–378, 1/200), IFNγ-APC (XMG-1.2, Biolegend, 1/200). For intracellular staining of Treg and Th1 cells, the FoxP3 staining kit (Miltenyi) and Cytofix Cytoperm (BD) were used, respectively, according to the manufacturer’s instructions. For extracellular staining of LPMC, CD11b-PECy7 (M1/70-BioLegend, 1/200), CD11c-PECF594 (HL3, BD Bioscience, 1/200), Ly6C-APC-Cy7 (HK1.4, BioLegend, 1/200), IA/IE-BV510 (M5/114.15.2, Biolegend, 1/200), F4–80-PE (BM8, Biolegend, 1/200) and NK1.1-FITC (PK136, Biolegend, 1/200) were incubated for 20 min at 4°C. Cells were then washed and fixed using PFA 2% solution diluted in PBS with EDTA at 2 mM. Stained samples were run on an Attune (Thermo Fischer) and eight-color flow cytometry analyses were performed with FlowJo software (Tree Star, USA). For intracellular cytokine staining, cells were stimulated for 5 h at 37°C with 50 ng/ml of phorbol 12-myristate 13-acetate (PMA; Calbiochem), 1 μg/ml of Ionomycin (Sigma), and 5 μg/mL of Brefeldin-A after 3 h of stimulation (BioLegend).

### Assessing BMDM and LPMC response to CBM588

Heat-killed vegetative CBM588 or butyric acid at a concentration of 0.2 mM or 1.5% of culture supernatant from CBM588 were added for 1 h at 37°C to LPMC or BMDCs cultured onto specific low binding 96 wells plates in complete RPMI Cells were then centrifuged and incubated in complete RPMI with CD4^+^ T cells or CD8^+^ T cells (or none as control). Co-cultures were set up at a ratio of 1 LPMC to 2 CD4^+^ T or 2 CD8^+^ T cells. The low endotoxin and azide-free purified IL-10 R – specific monoclonal antibody (clone 1B1.3A, Euromedex) was added at a concentration of 1 microg/mL for blocking IL-10 signaling. As a control, the rat IgG1 isotype was used. Culture supernatants were collected after overnight treatment.

### Cytokine measurements by ELISA

Serum and cell culture supernatant were collected for measuring levels of IFN-gamma and IL-10 by following the manufacturer’s instructions for ELISA (R&D Systems).

### 16S rRNA sequencing-based analysis of the gut microbiota

The procedure of feces sampling and the protocol of DNA extraction are described in the online supplementary material and methods section. The V3–V4 region of the 16S rRNA gene was amplified via a polymerase chain reaction (PCR) using barcoded primers, as previously reported.^37^ The TaKaRa Ex Taq Hot Start Version (Takara Bio Inc.) was used for PCR amplification, and the resulting PCR products were purified based on size selection using SPRI select (Beckman Coulter). DNA was quantified using a QuantiFluor ONE dsDNA System (Promega Corporation). Mixed samples were prepared by pooling approximately equal amounts of amplified and purified DNA, and these were sequenced using MiSeq Reagent Kit v3 (600 cycles) and a MiSeq sequencer according to the manufacturer’s instructions (Illumina). 16S rRNA gene sequence data obtained from the MiSeq sequencer were then processed using Quantitative Insights into Microbial Ecology (QIIME 2 2021. 2, http://qiime2.org/).^39^ An amplicon sequence variant (ASV) table was obtained by the demultiplexing and quality filtering of raw sequence data with the q2-demux plugin, followed by denoising with DADA2.^40^ The taxonomy of each variant was assigned at the species level by comparisons with the SILVA 138.1 rRNA database.^41^ To assess the within community diversity, α-diversity was examined using several metrics (Shannon index, Chao1 index, observed features, evenness, Simpson index, Simpson’s evenness measure E, Faith’s phylogenetic diversity), and the values of which were calculated using QIIME2. To assess distances between samples, β-diversity was estimated using the Bray-Curtis index, the Jaccard index, the weighted and the unweighted UniFrac metric and visualized using a principal coordinate analysis (PCoA). A linear discriminant analysis (LDA) Effect Size (LEfSe) was used with default settings to identify bacterial features that were differentially represented between the control and treatment groups.

### Statistics

Data were analyzed with either Prism 5 (GraphPad) or R software version 4.1.1 (The R Foundation for Statistical Computing, 2021-08-10). Data were depicted as means ± SEM. Groups of data were compared with two-way ANOVA or Mann–Whitney U test where applicable. For the microbiome data, Kruskal–Wallis test and *post-hoc* Mann–Whitney *U* test with Benjamini–Hochberg adjustment were used. All reported tests are two-tailed and differences were considered to be statistically significant when p-values < .05.

## Supplementary Material

Supplementary material clean.docx

## Data Availability

All data relevant to the study are included in the article or available as online supplemental information. 16S rRNA data have been deposited to the DNA Data Bank of Japan (accession number DRA017067).

## References

[1] Paz-Ares L, Luft A, Vicente D, Tafreshi A, Gumus M, Mazieres J, Hermes B, Çay Şenler F, Csőszi T, Fülöp A. et al. Pembrolizumab plus chemotherapy for squamous non–small-Cell lung cancer. N Engl J Med. 2018;379(21):2040–17. doi:10.1056/NEJMoa1810865. 30280635

[2] Reck M, Rodriguez-Abreu D, Robinson AG, Hui R, Csoszi T, Fulop A, Gottfried M, Peled N, Tafreshi A, Cuffe S. et al. Pembrolizumab versus chemotherapy for PD-L1–positive non–small-Cell lung cancer. N Engl J Med. 2016;375(19):1823–1833. doi:10.1056/NEJMoa1606774. 27718847

[3] Borghaei H, Paz-Ares L, Horn L, Spigel DR, Steins M, Ready NE, Chow LQ, Vokes EE, Felip E, Holgado E. et al. Nivolumab versus docetaxel in advanced nonsquamous non–small-Cell lung cancer. N Engl J Med. 2015;373(17):1627–1639. doi:10.1056/NEJMoa1507643. 26412456 PMC5705936

[4] Brahmer J, Reckamp KL, Baas P, Crino L, Eberhardt WE, Poddubskaya E, Antonia S, Pluzanski A, Vokes EE, Holgado E. et al. Nivolumab versus Docetaxel in Advanced Squamous-Cell Non–Small-Cell Lung Cancer. N Engl J Med. 2015;373(2):123–135. doi:10.1056/NEJMoa1504627. 26028407 PMC4681400

[5] Ellis PM, Vella ET, Ung YC. Immune checkpoint inhibitors for patients with advanced non–small-Cell lung cancer: a systematic review. Clin Lung Cancer. 2017;18(5):444–459.e1. doi:10.1016/j.cllc.2017.02.001. 28416123

[6] Borghaei H, Brahmer J. Nivolumab in Nonsquamous Non-Small-Cell Lung Cancer. N Engl J Med. 2016;374:493–494. 10.1056/NEJMc1514790 26840144

[7] Lurienne L, Cervesi J, Duhalde L, de Gunzburg J, Andremont A, Zalcman G, Buffet R, Bandinelli P-A. NSCLC immunotherapy efficacy and antibiotic use: a systematic review and meta-analysis. J Thorac Oncol. 2020;15(7):1147–1159. doi:10.1016/j.jtho.2020.03.002. 32173463

[8] Daillere R, Vetizou M, Waldschmitt N, Yamazaki T, Isnard C, Poirier-Colame V, Duong CM, Flament C, Lepage P, Roberti M. et al. Enterococcus hirae and Barnesiella intestinihominis facilitate cyclophosphamide-induced therapeutic immunomodulatory effects. Immunity. 2016;45(4):931–43. doi:10.1016/j.immuni.2016.09.009. 27717798

[9] Tomita Y, Ikeda T, Sakata S, Saruwatari K, Sato R, Iyama S, Jodai T, Akaike K, Ishizuka S, Saeki S. et al. Association of Probiotic Clostridium butyricum Therapy with survival and response to Immune Checkpoint Blockade in patients with lung cancer. Cancer Immunol Res. 2020;8(10):1236–1242. doi:10.1158/2326-6066.CIR-20-0051. 32665261

[10] Tomita Y, Goto Y, Sakata S, Imamura K, Minemura A, Oka K, Hayashi A, Jodai T, Akaike K, Anai M. et al. Clostridium butyricum therapy restores the decreased efficacy of immune checkpoint blockade in lung cancer patients receiving proton pump inhibitors. Oncoimmunology. 2022;11(1):2081010. doi:10.1080/2162402X.2022.2081010. 35655708 PMC9154751

[11] Dizman N, Meza L, Bergerot P, Alcantara M, Dorff T, Lyou Y, Frankel P, Cui Y, Mira V, Llamas M. et al. Nivolumab plus ipilimumab with or without live bacterial supplementation in metastatic renal cell carcinoma: a randomized phase 1 trial. Nat Med. 2022;28(4):704–12. doi:10.1038/s41591-022-01694-6. 35228755 PMC9018425

[12] Seki H, Shiohara M, Matsumura T, Miyagawa N, Tanaka M, Komiyama A, KURATA S. Prevention of antibiotic-associated diarrhea in children by Clostridium butyricum MIYAIRI. Pediatr Int. 2003;45(1):86–90. doi:10.1046/j.1442-200X.2003.01671.x. 12654076

[13] Hayashi A, Sato T, Kamada N, Mikami Y, Matsuoka K, Hisamatsu T, Hibi T, Roers A, Yagita H, Ohteki T. et al. A single strain of Clostridium butyricum induces intestinal IL-10-producing macrophages to suppress acute experimental colitis in mice. Cell Host Microbe. 2013;13(6):711–22. doi:10.1016/j.chom.2013.05.013. 23768495

[14] Kashiwagi I, Morita R, Schichita T, Komai K, Saeki K, Matsumoto M, Takeda K, Nomura M, Hayashi A, Kanai T. et al. Smad2 and Smad3 Inversely Regulate TGF-β Autoinduction in Clostridium butyricum-Activated Dendritic Cells. Immunity. 2015;43(1):65–79. doi:10.1016/j.immuni.2015.06.010. 26141582

[15] Li HY, McSharry M, Bullock B, Nguyen TT, Kwak J, Poczobutt JM, Sippel TR, Heasley LE, Weiser-Evans MC, Clambey ET. et al. The tumor microenvironment regulates sensitivity of murine lung tumors to PD-1/PD-L1 antibody blockade. Cancer Immunol Res. 2017;5(9):767–777. doi:10.1158/2326-6066.CIR-16-0365. 28819064 PMC5787226

[16] Blatner NR, Mulcahy MF, Dennis KL, Scholtens D, Bentrem DJ, Phillips JD, Ham S, Sandall BP, Khan MW, Mahvi DM. et al. Expression of RORγt marks a pathogenic regulatory T cell subset in human colon cancer. Sci Transl Med. 2012;4(164):164ra59. doi:10.1126/scitranslmed.3004566. PMC3762575 23241743

[17] Kedmi R, Najar TA, Mesa KR, Grayson A, Kroehling L, Hao Y, Hao S, Pokrovskii M, Xu M, Talbot J. et al. A RORγt+ cell instructs gut microbiota-specific Treg cell differentiation. Nature. 2022;610(7933):737–743. doi:10.1038/s41586-022-05089-y. 36071167 PMC9908423

[18] Sefik E, Geva-Zatorsky N, Oh S, Konnikova L, Zemmour D, McGuire AM, Burzyn D, Ortiz-Lopez A, Lobera M, Yang J. et al. Individual intestinal symbionts induce a distinct population of RORγ + regulatory T cells. Science. 2015;349(6251):993–997. doi:10.1126/science.aaa9420. 26272906 PMC4700932

[19] Fidelle M, Rauber C, Alves Costa Silva C, Tian AL, Lahmar I, de La Varende AM, Zhao L, Thelemaque C, Lebhar I, Messaoudene M. et al. A microbiota-modulated checkpoint directs immunosuppressive intestinal T cells into cancers. Science. 2023;380(6649):eabo2296. doi:10.1126/science.abo2296. 37289890

[20] Derosa L, Routy B, Thomas AM, Iebba V, Zalcman G, Friard S, Mazieres J, Audigier-Valette C, Moro-Sibilot D, Goldwasser F. et al. Intestinal Akkermansia muciniphila predicts clinical response to PD-1 blockade in patients with advanced non-small-cell lung cancer. Nat Med. 2022;28(2):315–24. doi:10.1038/s41591-021-01655-5. 35115705 PMC9330544

[21] Sato R, Tanaka M. Intestinal distribution and intraluminal localization of orally administered Clostridium butyricum in rats. Microbiol Immunol. 1997;41(9):665–71. doi:10.1111/j.1348-0421.1997.tb01909.x. 9343816

[22] Liu W, Ma F, Sun B, Liu Y, Tang H, Luo J, Chen H, Luo Z. Intestinal microbiome associated with immune-related adverse events for patients treated with anti-PD-1 inhibitors, a real-world study. Front Immunol. 2021;12:756872. doi:10.3389/fimmu.2021.756872. 34975845 PMC8716485

[23] Yang BY, Zhao FZ, Li XH, Zhao MS, Lv JC, Shi MJ, Li J, Zhou Z-Y, Wang J-J, Song J. et al. Alteration of pro-carcinogenic gut microbiota is associated with clear cell renal cell carcinoma tumorigenesis. Front Microbiol. 2023;14:1133782. doi:10.3389/fmicb.2023.1133782. 37089532 PMC10113506

[24] Ohnmacht C, Park JH, Cording S, Wing JB, Atarashi K, Obata Y, Gaboriau-Routhiau V, Marques R, Dulauroy S, Fedoseeva M. et al. The microbiota regulates type 2 immunity through RORγt + T cells. Science. 2015;349(6251):989–993. doi:10.1126/science.aac4263. 26160380

[25] Baban B, Chandler PR, Sharma MD, Pihkala J, Koni PA, Munn DH, Mellor AL. IDO activates regulatory T cells and blocks their conversion into Th17-like T cells. J Immunol. 2009;183(4):2475–2483. doi:10.4049/jimmunol.0900986. 19635913 PMC3677163

[26] Sharma MD, Baban B, Chandler P, Hou DY, Singh N, Yagita H, Azuma M, Blazar BR, Mellor AL, Munn DH. et al. Plasmacytoid dendritic cells from mouse tumor-draining lymph nodes directly activate mature tregs via indoleamine 2,3-dioxygenase. J Clin Invest. 2007;117(9):2570–82. doi:10.1172/JCI31911. 17710230 PMC1940240

[27] Rankin LC, Kaiser KA, de Los Santos-Alexis K, Park H, Uhlemann AC, Gray DHD, Arpaia N. Dietary tryptophan deficiency promotes gut RORγt+ Treg cells at the expense of Gata3+ Treg cells and alters commensal microbiota metabolism. Cell Rep. 2023;42(3):112135. doi:10.1016/j.celrep.2023.112135. 36840944 PMC10150404

[28] Qiao M, Zhou F, Liu X, Jiang T, Wang H, Jia Y, Li X, Zhao C, Cheng L, Chen X. et al. Interleukin-10 induces expression of CD39 on CD8+T cells to potentiate anti-PD1 efficacy in EGFR-mutated non-small cell lung cancer. J Immunother Cancer. 2022;10(12):10. doi:10.1136/jitc-2022-005436. PMC9772697 36543373

[29] Qiao J, Liu Z, Dong C, Luan Y, Zhang A, Moore C, Fu K, Peng J, Wang Y, Ren Z. et al. Targeting tumors with IL-10 Prevents Dendritic Cell-Mediated CD8(+) T Cell apoptosis. Cancer Cell. 2019;35(6):901–915.e4. doi:10.1016/j.ccell.2019.05.005. 31185213

[30] Lee GK, Park HJ, Macleod M, Chandler P, Munn DH, Mellor AL. Tryptophan deprivation sensitizes activated T cells to apoptosis prior to cell division. Immunology. 2002;107(4):452–60. doi:10.1046/j.1365-2567.2002.01526.x. 12460190 PMC1782830

[31] Habtezion A, Nguyen LP, Hadeiba H, Butcher EC. Leukocyte trafficking to the small intestine and colon. Gastroenterology. 2016;150(2):340–54. doi:10.1053/j.gastro.2015.10.046. 26551552 PMC4758453

[32] Bliss SK, Bliss SP, Beiting DP, Alcaraz A, Appleton JA. IL-10 regulates movement of intestinally derived CD4+ T cells to the liver. J Immunol. 2007;178(12):7974–83. doi:10.4049/jimmunol.178.12.7974. 17548634

[33] Yurchenko E, Shio MT, Huang TC, Da Silva Martins M, Szyf M, Levings MK, Olivier M, Piccirillo CA. Inflammation-driven reprogramming of CD4+ Foxp3+ regulatory T cells into pathogenic Th1/Th17 T effectors is abrogated by mTOR inhibition in vivo. PloS One. 2012;7(4):e35572. doi:10.1371/journal.pone.0035572. 22545118 PMC3335853

[34] Hagihara M, Yamashita R, Matsumoto A, Mori T, Kuroki Y, Kudo H, Oka K, Takahashi M, Nonogaki T, Yamagishi Y. et al. The impact of Clostridium butyricum MIYAIRI 588 on the murine gut microbiome and colonic tissue. Anaerobe. 2018;54:8–18. doi:10.1016/j.anaerobe.2018.07.012. 30076897

[35] Ariyoshi T, Hagihara M, Eguchi S, Fukuda A, Iwasaki K, Oka K, Takahashi M, Yamagishi Y, Mikamo H. Clostridium butyricum MIYAIRI 588-induced protectin D1 has an anti-inflammatory effect on antibiotic-induced intestinal disorder. Front Microbiol. 2020;11:587725. doi:10.3389/fmicb.2020.587725. 33193245 PMC7661741

[36] Kanai T, Mikami Y, Hayashi A. A breakthrough in probiotics: Clostridium butyricum regulates gut homeostasis and anti-inflammatory response in inflammatory bowel disease. J Gastroenterol. 2015;50(9):928–39. doi:10.1007/s00535-015-1084-x. 25940150

[37] Hagihara M, Kuroki Y, Ariyoshi T, Higashi S, Fukuda K, Yamashita R, Matsumoto A, Mori T, Mimura K, Yamaguchi N. et al. Clostridium butyricum modulates the microbiome to protect intestinal barrier function in mice with Antibiotic-Induced Dysbiosis. iScience. 2020;23(1):100772. doi:10.1016/j.isci.2019.100772. 31954979 PMC6970176

[38] Bain CC, Bravo-Blas A, Scott CL, Perdiguero EG, Geissmann F, Henri S, Malissen B, Osborne LC, Artis D, Mowat AM. et al. Constant replenishment from circulating monocytes maintains the macrophage pool in the intestine of adult mice. Nat Immunol. 2014;15(10):929–37. doi:10.1038/ni.2967. 25151491 PMC4169290

[39] Bolyen E, Rideout JR, Dillon MR, Bokulich NA, Abnet CC, Al-Ghalith GA, Alexander H, Alm EJ, Arumugam M, Asnicar F. et al. Reproducible, interactive, scalable and extensible microbiome data science using QIIME 2. Nat Biotechnol. 2019;37(8):852–7. doi:10.1038/s41587-019-0209-9. 31341288 PMC7015180

[40] Callahan BJ, McMurdie PJ, Rosen MJ, Han AW, Johnson AJ, Holmes SP. DADA2: High-resolution sample inference from Illumina amplicon data. Nat Methods. 2016;13(7):581–3. doi:10.1038/nmeth.3869. 27214047 PMC4927377

[41] Quast C, Pruesse E, Yilmaz P, Gerken J, Schweer T, Yarza P, Peplies J, Glöckner FO. The SILVA ribosomal RNA gene database project: improved data processing and web-based tools. Nucleic Acids Res. 2013;41(D1):D590–6. doi:10.1093/nar/gks1219. 23193283 PMC3531112

