# Supplementary material for: The biotherapeutic Clostridium butyricum MIYAIRI 588 strain potentiates enterotropism of Rorγt+Treg and PD-1 blockade efficacy
Source: Gut Microbes. 2024 Feb 22;16(1):2315631. doi: 10.1080/19490976.2024.2315631 (PMC10885180; doi:10.1080/19490976.2024.2315631)
Supplement: Supplementary material clean.docx [file KGMI_A_2315631_SM9675.docx]

**The biotherapeutic *Clostridium butyricum* MIYAIRI 588 strain potentiates**

**enterotropism of Ror**γ **t+Treg and PD-1 blockade efficacy.**

Thomas Paz Del Socorro1, Kentaro Oka2, Olivier Boulard1, Motomichi Takahashi2,

Lionel Franz Poulin1, Atsushi Hayashi2,*, Mathias Chamaillard1,*

1 University of Lille, Inserm, U1003, F-59000 Lille, France.

2 R&D Division, Miyarisan Pharmaceutical Co., Ltd., Saitama, Japan.

*** co-corresponding authors**

[mathias.chamaillard@inserm.fr](mailto:mathias.chamaillard@inserm.fr) and [a.hayashi@miyarisan.com](mailto:a.hayashi@miyarisan.com)

**Supplementary figure 1: Assessment of the responsiveness to PD-1 blockade**

**based on the individual growth rate after a week of treatment.** The number of

responder (R) mice out of 5 is indicated for two independent experiments of 5 mice per

group.

**Supplementary figure 2: Assessment of intestinal permeability upon PD-1**

**blockade.** C57BL/6J mice were administered either anti-PD-1 or isotype control mAbs

when implanted tumors reached a minimum surface of 40mm2. Two days later, mice

were water-starved overnight. Mice were then orally administered FITC-dextran at the

beginning of the following day. (A) Measurements of the blood concentration of FITC

dextran. (B) The same procedure was carried out in a separate group of mice that had

received Imipenem. (C-D) RT-qPCR analysis of Zo1 expression in the colon and the

ileum (n=5/group).

**Supplementary figure 3: Impact of CBM588 supplementation on the immune**

**contexture of the spleen from tumor-bearing mice.** (A) Splenic frequency of IFNγ -

expressing CD8+ T cells (Tc1). (B) Frequency of Tbet-expressing CD4+ cells in spleen,

(C) Frequency of CD25+Foxp3+ Treg cells in spleen, (D) Frequency of RorγT-expressi ng

Treg cells in spleen. A representative experiment containing 5 mice/group out of two is

depicted. Data are plotted as means ± SEM and P values were calculated using the

Mann-Whitney U test.

**Supplementary figure 4: The response rate is not markedly influenced by the**

**bacterial community variation within sample and the dissimilarities between**

**samples.** (A) Boxplots representing multiple metrics of alpha diversity from tumor-

bearing mice at day 14. (B) PCoA plots of several metrics of beta diversity from tumor-

bearing mice at day 14.

**Supplementary figure 5: The Ror**γ **t-expressing Treg is more abundant in the colon**

**than the ileum.** A representative contour plot of the ileal and colonic Rorγt-expressing

Treg cells is depicted.

**Supplementary figure 6: Impact of CBM588 supplementation on the intestinal**

**expression of *genes* involved in regulation of regulatory T cells homing and**

**development.** RT-qPCR analysis of gene expression from the large intestine. Each

dot represent a biological replicate (n=5/group) accordingly to legend colors. A

representative experiment with 5 mice per group out of two yielding similar results of

RT-qPCR analysis is depicted.

**Supplementary figure 7: CBM588 enhances the expression of IDO-1 in bone-**

**marrow derived dendritic cells.** Quantitative measurements by qRT-PCR analysis of

IDO-1 in bone marrow derived dendritic cells that were treated at different dose of heat-

killed CBM588.

**Supplementary figure 8: CBM588 promotes the secretion of Interleukin-10 by**

**bone marrow derived macrophages and lamina propria mononuclear cells.**

Quantitative measurements by ELISA of IL10 in the supernatant from bone marrow

derived macrophages and lamina propria mononuclear cells that were treated at

different dose of heat-killed CBM588.

**Supplementary figure 9: Supplementation with live CBM588 promotes**

**immunogenic conversion of the tumor microenvironment.** RT-qPCR from tumor

tissues of two independent experiments (n=10/group of each experiment except for the

Combo group in which one tumor was excluded because of being necrotic). P values

were calculated using the Mann-Whitney U test.

**Supplementary figure 10: Treatment of LPMC with CBM588 culture supernatant**

**fails to modulate the secretion of interleukin-10.** Quantitative measurements of

Interleukin-10 in the supernatant from LPMCs from tumor bearers treated with culture

supernatant of CBM588 (at a dilution of 1/66) upon or not blockade of IL-10 signaling.

P values were calculated using the Mann-Whitney U test.

**Exp 1 Exp 2**

**1250**


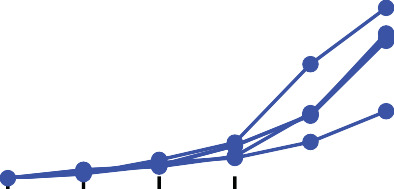


**Iso**

**Tumor volume (mm^3^)**

**Tumor volume (mm^3^)**

**1000**

**750**

**500**

**250**

**0**

**1250**


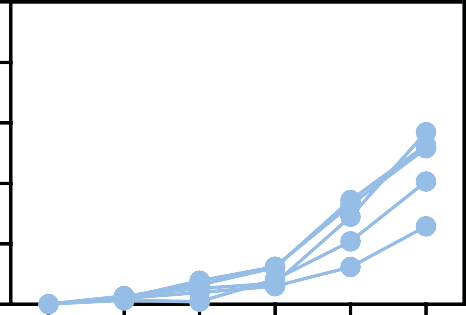


**CBM588 (2 R/5)**

**1000**

**750**

**500**

**250**

**0**

**0 5 7 10 12 14**

**Tumor volume (mm^3^)**

**Days post inoculation**

**0 5 7 10 12 14**

**Days post inoculation**


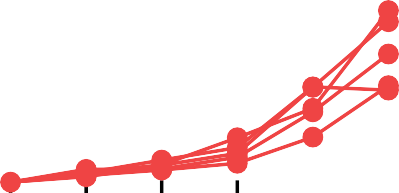


**aPD1 (1 R/5)**

**0 5 7 10 12 14**

**Days post inoculation**


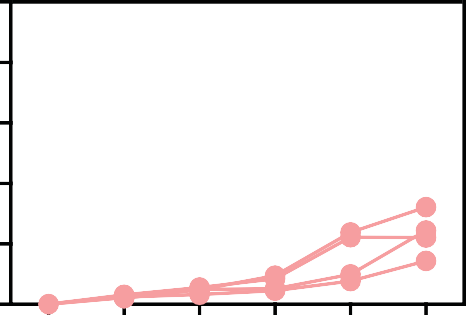


**CBM588 + aPD1 (4 R/4)**

**0 5 7 10 12 14**

**Days post inoculation**

**1250**

**1000**


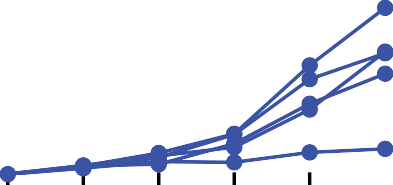


**Iso**

**750**

**500**

**250**

**0**

**1250**


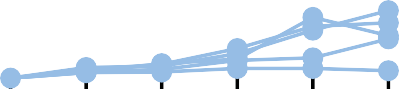


**CBM588 (4 R/5)**

**Tumor volume (mm^3^)**

**1000**

**750**

**500**

**250**

**0**

**0 5 7 10 12 14**

**Days post inoculation**

**0 5 7 10 12 14**

**Days post inoculation**


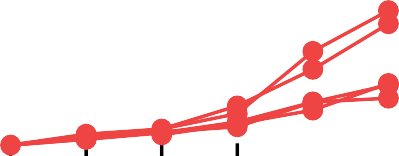


**aPD1 (1 R/5)**

**0 5 7 10 12 14**

**Days post inoculation**


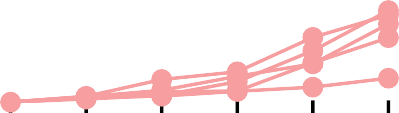


**CBM588 + aPD1 (2 R/5)**

**0 5 7 10 12 14**

**Days post inoculation**

**A B**


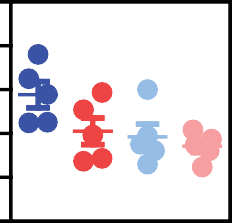
**1.0**

**FITC-dexran (µg/mL)**

**0.8**

**0.6**

**0.4**

**0.2**

**Colon**

**3**


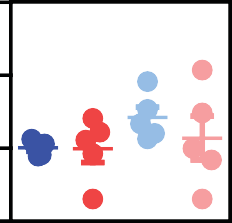


***Zo-1***

**Fold change**

**2**

**1**

**0.0**

**aPD-1**

**- + - +**

**0**

**aPD-1**

**- + - +**

## CBM588 -

**- + +**

## CBM588 -

**- + +**

**A B**


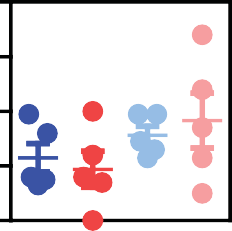
**0.8**

**0.6**

**Tc1 (%)**

**0.4**

**0.2**

**0.0**

**Spleen**

**C D**

**CD25^+^Foxp3^+^(%)**


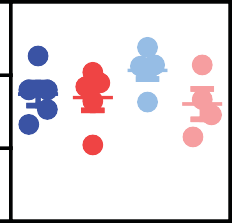

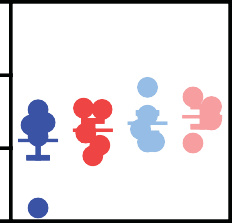

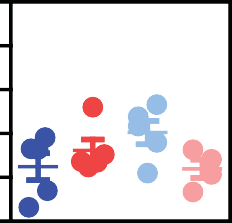
**5 15 15**

**Tbet^+^CD4^+^ (%)**

**Ror**γ**T^+^T_reg_ (%)**

**4**

**3 10 10**


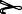
**2 5 5**

**1**

**0 0 0**

**aPD-1 - + - +**

**aPD-1 - + - +**

**aPD-1 - + - +**

**aPD-1 - + - +**

## CBM588

**- - + +**

## CBM588

**- - + +**

## CBM588

**- - + +**

## CBM588

**- - + +**

**A**

**400**


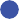


**350**

**300**

**250**

**200**

**150**

# Chao1

**Iso NR R**

**7.0**

**6.5**

**6.0**

**5.5**

**5.0**

# Shannon

**Iso NR R**


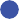


**0.80**

**0.75**

**0.70**

**0.65**

# Evenness

**Iso NR R**

# Observed features


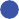


**350**

**300**

**250**

**200**

**150**

**Iso NR R**

# B Unweighted unifrac

**0.06**


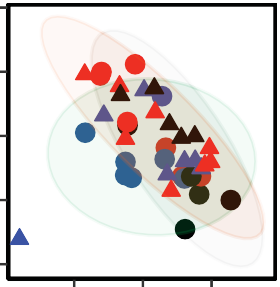


***p<0.05***

**0.03**

**PC2 (7%)**


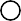
**0.00**

**-0.03**

**-0.06**

**18**

**16**

**14**

**1st 2nd**

# Faith pd

**Iso NR R**


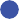

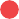


**Experiment**

**0.150**

**0.125**

**0.100**

**0.075**

**0.050**

**0.05**


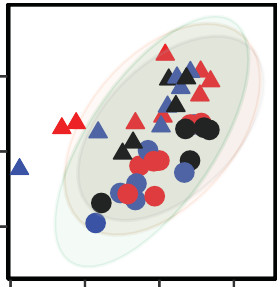


***p=0.02***

**PC2 (6.9%)**

**0.00**

**-0.05**

# Simpson e

**Iso NR R**

***p=0.04***

# Jaccard

**0.3**

**PC2 (27.3%)**

**0.2**

**0.1**

**0.0**

**-0.1**

**0.24 0.28 0.32**

**PC1 (50.8%)**

# Weighted unifrac

**Isotype (Iso)**


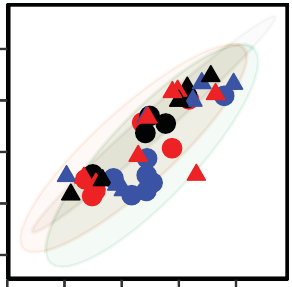


**Non responder (NR) Responder (R)**

**0.20 0.25 0.30 0.35**

**PC1 (31.6%)**

# Bray curtis

**0.10**


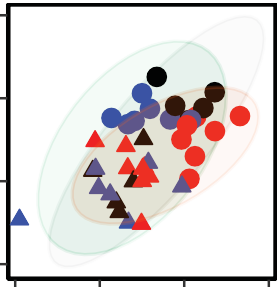


***p<0.04***

**PC2 (10.1%)**

**0.05**

**0.00**

**-0.05**

**-0.3 -0.2-0.1 0.0 0.1**

## PC1 (42.1%)

**0.25 0.30 0.35 0.40**

## PC1 (38.6%)

**Colon lleon**

**Foxp3**


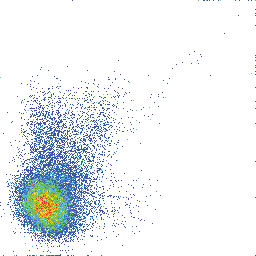


3.08


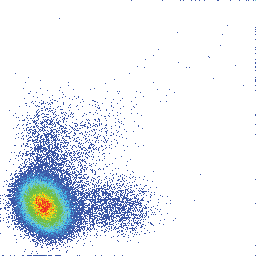


**0.66**

**Rorgt**


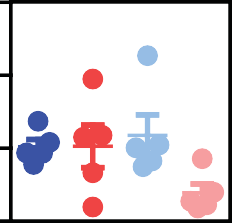

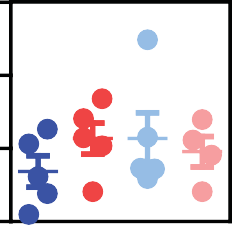

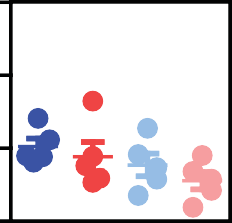

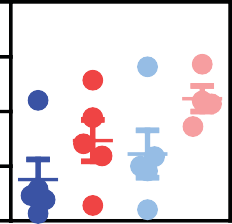

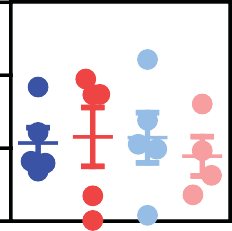
**3**

**Fold change**

| ***Tbx21*** | **6** | ***Icos*** | **3** | ***Tnfa*** | **8** | ***GrzmB*** | **3** | ***Ifng*** |
| --- | --- | --- | --- | --- | --- | --- | --- | --- |
|  | **4** |  | **2** |  | **6** |  | **2** |  |
|  |  |  |  |  | **4** |  |  |  |
|  | **2** |  | **1** |  | **2** |  | **1** |  |
|  |  | | | | | | | |

**2**

**1**

**0**

| **- +** | **-** | **+** | **0**  **- +** | **-** | **+** | **0**  **- +** | **-** | **+** | **0**  **- +** | **-** | **+** | **0**  **- +** | **-** | **+** |
| --- | --- | --- | --- | --- | --- | --- | --- | --- | --- | --- | --- | --- | --- | --- |
| **- -** | **+** | **+** | **- -** | **+** | **+** | **- -** | **+** | **+** | **- -** | **+** | **+** | **- -** | **+** | **+** |

**aPD-1**

## CBM588

| ***Madcam1*** | **6**  **4**  **2** | ***Vdr*** | **3**  **2**  **1** | ***Ahr*** | **4**  **3**  **2**  **1** | ***Ffar2*** | **25**  **20**  **15**  **10**  **5** | ***Reg3b*** |
| --- | --- | --- | --- | --- | --- | --- | --- | --- |
|  | **0** |  | **0** |  | **0** |  | **0** |  |


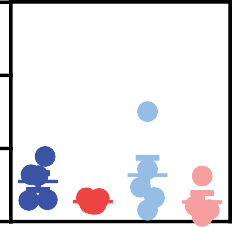

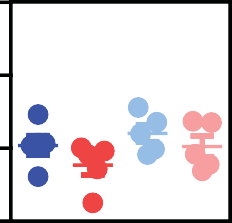

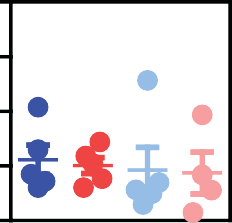

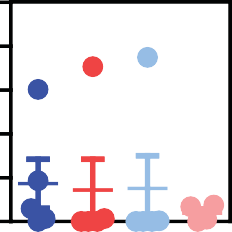

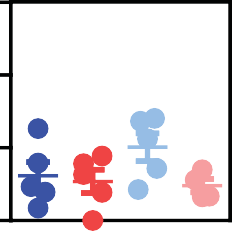
**6**

**Fold change**

**4**

**2**

**0 aPD-1**

| **- +** | **-** | **+** | **- +** | **-** | **+** | **- +** | **-** | **+** | **- +** | **-** | **+** | **- +** | **-** | **+** |
| --- | --- | --- | --- | --- | --- | --- | --- | --- | --- | --- | --- | --- | --- | --- |
| **- -** | **+** | **+** | **- -** | **+** | **+** | **- -** | **+** | **+** | **- -** | **+** | **+** | **- -** | **+** | **+** |

## CBM588

**10**

**Relative Ido1 expression (fold)**

**8**

**6**

**4**

**2**

**0**

**0**

**1**

Heat-killed CBM588

**A**

**1500**


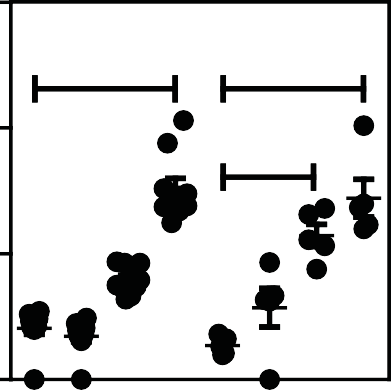


***

***

**

**1000**

**IL-10 (pg/ml)**

**500**

**0**

**Heat-killed CBM 0 0.1 1 10 0 0.1 1 10**

**B**

**600**

## BMDM

**LPMCs**

**400**


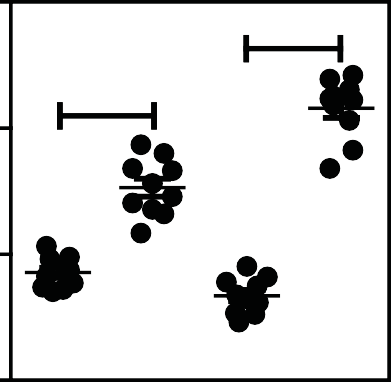


***

*

**IL-10 (pg/ml)**

**200**

**0**

**Heat-killed CBM 1 10 1 10**

## BMDM BMDC

| **aPD-1** | **- +** | **-** | **+** | **- +** | **-** | **+** | **- +** | **-** | **+** | **- +** | **-** | **+** | **- +** | **-** | **+** | **- +** | **-** | **+** | **- +** | **-** | **+** |
| --- | --- | --- | --- | --- | --- | --- | --- | --- | --- | --- | --- | --- | --- | --- | --- | --- | --- | --- | --- | --- | --- |
| **CBM588** | **- -** | **+** | **+** | **- -** | **+** | **+** | **- -** | **+** | **+** | **- -** | **+** | **+** | **- -** | **+** | **+** | **- -** | **+** | **+** | **- -** | **+** | **+** |


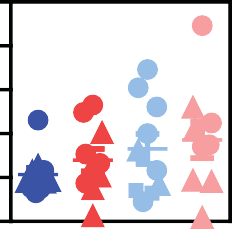
**Tumor**

| **30**  **20**  **10** | ***Ifng*** 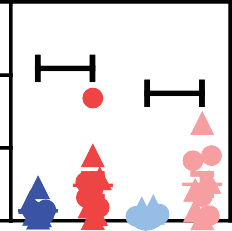 * | ** | **40**  **30**  **20**  **10** | ***Cxcl9*** 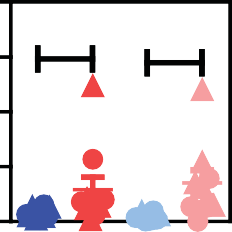 * | ** | **200**  **150**  **100**  **50** | ***Ido1*** 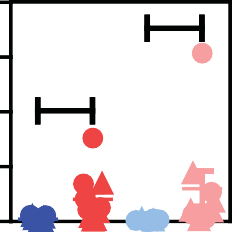 ** | *** | **6**  **4**  **2** | ***Icos*** 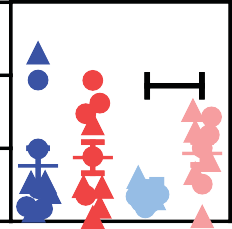 | ** | **10**  **8**  **6**  **4**  **2** | ***Grzmb*** 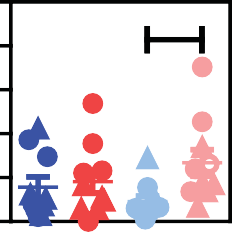 | ** | **6**  **4**  **2** | ***Ccl2*** 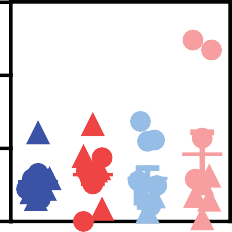 | **5**  **4**  **3**  **2**  **1** | ***Tnfa*** |
| --- | --- | --- | --- | --- | --- | --- | --- | --- | --- | --- | --- | --- | --- | --- | --- | --- | --- | --- |
| **0** |  |  | **0** |  |  | **0** |  |  | **0** |  |  | **0** |  |  | **0** |  | **0** |  |

**100**


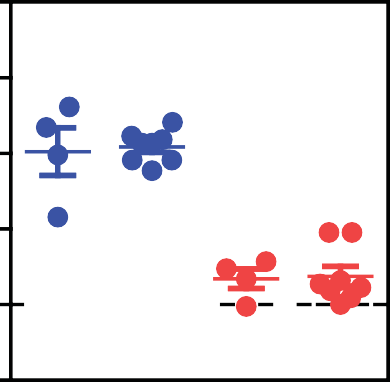


**80**

**IL-10 (pg/ml)**

**60**

**40**

**20**

**0**

**Supernatant CBM -**

**+ - +**


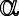
**Isotype IL10R** α
